# Experiences of Early and Enhanced Clinical Exposure for Postgraduate Neonatal Nursing Students at the University of Zambia, School of Nursing Sciences: Lessons and Implications for the Future

**DOI:** 10.4236/ojn.2023.136024

**Published:** 2023-06-08

**Authors:** Victoria Mwiinga Kalusopa, Patricia Katowa-Mukwato, Kabwe Chitundu, Manasseh Mvula, Selestine Nzala, Marjorie Kabinga-Makukula, Christabell Mwiinga, Emmanuel Musenge Mwila, Linda Kampata, Micheal Kanyanta Mumba, Micheal Chiguntap, James Sichone, Concept Kwaleyela, Phadaless Phiri, Suzan Mutemwa, Mildred Zulu, Chileshe Mwaba-Siwale, Ruth Wahila, Mukumbuta Nawa, Mercy Monde Wamunyima, Francina Makondo, Charity Syatalimi, Elliot Kafumukache, Fastone Goma

**Affiliations:** 1 School of Nursing Sciences, University of Zambia, Lusaka, Zambia; 2 School of Medicine and Health Sciences, University of Lusaka, Lusaka, Zambia; 3 School of Medicine, University of Zambia, Lusaka, Zambia; 4 School of Public Health, University of Zambia, Lusaka, Zambia; 5 School of Health Sciences, University of Zambia, Lusaka, Zambia; 6 School of Medicine and Health Sciences, Mulungushi University, Kabwe, Zambia; 7 School of Public Health and Environmental Studies, Levy Mwanawasa Medical University, Lusaka, Zambia; 8 Medical Library, University of Zambia, Lusaka, Zambia; 9 Main Library, University of Zambia, Lusaka, Zambia

**Keywords:** Experience, Clinical Exposure, Postgraduate, Early, Enhanced

## Abstract

**Background and Objectives:**

Early and Enhanced Clinical Exposure immediately places postgraduate students in a clinical setting and incorporates continual hands-on instruction throughout their studies. It aims to motivate students by strengthening their academics, improving clinical and communication skills, and increasing their confidence. The underlying principles are to provide a clinical context and to ensure that the patient remains the centre of learning. The School of Nursing Sciences implemented this model in 2021 to produce hands-on Masters-level neonatal practitioners who can work in multidisciplinary clinical contexts. Therefore, this study explored the experiences of postgraduate nursing students on the Early and Enhanced Clinical Exposure model and draw implications for the future.

**Methods:**

A phenomenological study design was utilized at the University of Zambia, School of Nursing Sciences and comprised of eight Master of Science Neonatal Nursing students in their second year. Convenience sampling was used to select the study site and participants. Data was collected between 15^th^ January 2023 and 31^st^ January 2023 using an in-depth interview guide. Audio recording and notes were transcribed immediately after data collection. Data analysis was conducted using thematic analysis and codes and themes were constructed from the coded data. Ethical clearance and permission were sought before conducting the study.

**Results:**

Four major themes emerged from the study: identity and role confusion, challenging and hectic experiences, positive educational experiences, and personal and professional growth. These themes contributed to the promotion of evidence-based practice by helping students to assess, diagnose, and treat various conditions, as well as gain interest, experience, knowledge, and exposure.

**Conclusion:**

The model has a significant impact on motivation to learn, as evidenced by reported increased skill level with potential for use in clinical practice. It is recommended that it be implemented in all postgraduate programs for full-time students.

## 1. Background

Early and Enhanced Clinical Exposure (EECE), as defined by [1], is a ground-breaking concept built on the premise of immediately integrating postgraduate students into the clinical context for continued hands-on learning throughout the training period. This teaching and learning model seeks to inspire students by fostering academic excellence, clinical and communication skill development, and increased self-assurance [2]. Most crucially, the system exposes postgraduate students to patients at a time when they are still developing in their training [3]. There are two (2) basic underlying principles of EECE namely: providing a clinical context, and ensuring that the patient remains the centre of learning [3]. Through this way, EECE provides an early clinical correlation to content learnt in the classroom, provides authentic human contact in a socio-clinical context, and lastly introduces humanities in medical sciences.

The three (3) situations in which EECE can take place were detailed by [2]. The most basic is the classroom where readymade case scenarios and clinical material like patient’s case notes can be discussed. The second and most important setting is the hospital which ensures that there is linkage between theory and understanding of cases, including signs, symptoms, and diagnosis. In addition, the hospital setting incorporates other sessions such as observation of student-patient relationship and empathy. For EECE to be effective in this setting, the cases should be discussed with students, and their context was linked to what was previously taught in the classroom. It also requires that students learn and finalize the topics related to cases before students’ placement. The third setting is the community which provides an ideal environment for teaching preventive medicine, ethics, health education and behavioural sciences in real time. In addition, this setting brings the socio-clinical relevance and context to the postgraduate students. Taking advantage of the effective influence EECE has on learning, the University of Zambia School of Nursing Sciences (UNZASoNS) devised the model in 2021 [1] for its postgraduate programs. The model was aimed at producing Masters-level Nurses and Midwifery Practitioners who are hands-on and more flexible to work in a multidisciplinary clinical context rather than focusing on teaching jobs as has been the case in the past [1]. In addition, EECE helps postgraduate nurses and midwives discover and conduct clinical research in order to provide evidence-based knowledge from the local context and address local nursing problems.

In as much as the EECE model has been widely thought to contribute immensely to the creation of opportunities for the development of empathy, as well as communication and practical skills in the students as described by [4], it is not infallible. [5] Argued that learning in the context of EECE is highly informal and includes a socialization process in which some learning outcomes may be valued or neglected. This study, therefore, explored the experiences of the postgraduate nursing students on the EECE model and further drew lessons and implications for the future. In addition, from the time the model was implemented by UNZASoNS, little is known about the experiences of the postgraduate nursing students and lectures that are using this model. The subject of early clinical exposure has become important during these times as the education system predominantly relied on allowing students to take their theory sessions first and then proceed to the clinical practice [6]. However, it was not until recently that the Early and Enhanced Clinical Exposure (EECE) model has been developed to cultivate medical professionalism among students [3]. This model has been adopted by some medical schools worldwide to close the gap between basic and clinical sciences [6].

Published literature has indicated that postgraduate students need an understanding of basic sciences and also require grounding in human and social aspects of their practice [3]. EECE model therefore enables the postgraduate students to be hands-on and more flexible to work in a multidisciplinary clinical context [1]. This is because the model has the potential to improve motivation for learning, promote deep learning, better understanding and longer retention of the knowledge gained, thus aiding in the effective learning of clinical skills [7]. On students’ experiences with EECE, there is the paucity of published literature as this concept is still in its infancy in Zambia and throughout Africa. The majority of the literature solely addressed the model’s use and effects at medical schools. Therefore, this study was the first to locally explore postgraduate nursing students’ experiences of early and enhanced clinical practice.

Ref. [8] strongly recommended integration-based practices such as early exposure of students to clinical discipline in medical related education [9]. In order for Early and Enhanced Clinical Exposure to be implemented effectively, there is a need for proper communication and feedback between the schools (including the teaching hospital where the students are allocated) and the students [10]. This requires coordinated efforts by the preclinical, paraclinical and clinical faculty. The students were exposed to the clinical area for half a day from Monday to Friday in the second and third terms. However, little was known about the student’s experiences prior to the exposure and after. Further, it might help the postgraduate students develop professionally, interact with patients with more confidence and less stress, and develop self-reflection and professional identity.

## 2. Materials and Methods

A phenomenological study design was utilized to bring out and describe the actual experiences of students with the EECE model. This model provides a clinical context for the postgraduate students to learn in and ensures that the patient remains the focal point of learning when they are still maturing in their training. Convenience sampling was used to select the study site and participants at UNZASoNs. Study population comprised of eight postgraduate students studying Neonatal nursing in their second year with no screening criteria other than meeting the inclusion criteria. This is so because the group, which consists of eight second-year students, is the first to employ the EECE model. Convenience sampling was used to select both the study site and participants for the study Sample.

Data collection was conducted from 15^th^ January 2023 to 31^st^ January 2023 using an in-depth interview guide. To avoid loss of data, the audio recording and notes about how each discussion took place were written immediately after data collection. Data was analysed using thematic analysis and the data was coded and themes were constructed from the coded data. Only second year students in the right frame of mind and gave consent for interviews after reading the information sheet were included in the study. Those who were not available or who were physically or mentally unstable at the time of the study were both grounds for exclusion. Each discussion started with an open-ended question and recorded appropriately for use during data analysis. The discussion notes that emanated from data transcriptions and audio recordings where locked in a cabinet which was only accessible to the researchers. Pseudonym identification codes were assigned to participants. For data security, the typed data sets were stored on a Password protected personal computer known only by the researchers.

Trustworthiness was achieved through maintenance of credibility, transferability, dependability and Confirmability. Credibility was ensured through use of direct participant quotes from the in-depth interview guide. Transferability ensured thoroughly describing the context of the study and how the themes emerged from the data collected. Dependability was achieved by providing the audio recordings of the in-depth interviews. Confirmability was ensured through maintenance of an audit trail. The audit trail provided evidence of decisions and choices made by the researchers regarding theoretical and methodological issues throughout the study with clear rationale.

## 3. Results

Four major themes emerged from the in-depth interviews, with 6 related subthemes (Table 1). During the analysis the collected data was converted from audio data to text data that discussed the experiences of MSc postgraduate students with EECE. Most of the discussion was revolving around Identity and role confusion, learning experience and enhancing personal growth.

### 3.1. Theme 1: Identity and Role Confusion

The theme identity and role confusion explained the confusion the students experienced as they went to the practice area. This theme was informed by two sub themes namely, identity confusion and emotional outburst.

#### Identity Confusion

The subtheme “identity confusion” reflected the misunderstandings that the students had with the way the programme was being coordinated. Most of the participants reported that there was no clear description of what they were expected to do while in the clinical area. They also stated that the objectives were not clearly spelt out it was somehow difficult for the students to place themselves and know their exact role as they practiced. As a result, some of participants started participating in the general routines followed by the nurses in the wards.

“I was not sure of what I needed to do on the ward and my role so when the nurses are doing top and tail or feeding I would also join”.

The confusion was further intensified by the mismatch between the objectives they had received from the school and the manner in which they were being executed in the clinical practice. They further, reported that most of the teaching in the clinical area was done by the medical doctors as a result most of the times the students would learn the content and remain wondering what their role is at this level. “*The doctor would explain the pathophysiology of a condition so well and teach how to make a diagnosis, but then I kept wondering what my nursing role is now that I know the pathophysiology*”.

These factors brought confusion in how the students viewed themselves and disturbed their professional identify as neonatal nurses.

#### Emotional Outburst

The subtheme “emotional outburst” reflected the emotional challenges the students experienced while in the clinical area. Most of the participants reported that they experienced some fear, anxiety and stress in the initial phase as they were exposed to the clinical area. From the report, this could have been due to the inadequate preparation they hard before coming to the clinical area.

“I was not fully prepared of my expectations at the clinical area”.

Some of the participants stated that they were afraid of practicing on the preterm babies who were too small for fear of making a mistake while handling the baby and consequently causing a death. Additionally, they reported that it was hard to practice for fear of receiving negative feedback from the doctors who were teaching them or appearing like they had no knowledge in the presence of other students.

“Because we were combined with the Medical students, I was afraid to make a mistake and look like I have no knowledge”.

On the other hand, the students reported that the hostile behaviour from the nurses made things worse as they were not welcoming or supportive. They reported that the nurse’s behaviour was rather disappointing because they were not particularly hospitable to them. Most participants reported that the nurse’s attitude towards the students was hostile because they thought the students were going to get their positions once they completed their studies. Also, the nurses did not see any difference in scope of practice between them and the postgraduate students.

“Sometimes when you ask nurses questions they would respond or show you were you need to find equipment to use as you care for the patients”.

The unsupportive behaviour by the nurses was a source of concern and very demotivating for the students as they thought how they would work with these nurses once we complete our programme.

Furthermore, the participants reported that sometimes the negative attitude from the nurses was as a result of the shortage in the staff working in the wards. They were also overwhelmed with number of the patients they had to attend to because the nurse patient ratio was so high. As a result they felt they needed to meet the patients’ needs first but in the process neglecting the students who needed their help.

### 3.2. Theme 2: Challenging and Hectic Experience

The theme challenging learning experience elucidated the negatives experiences that the students had with regards to the use of the EECE model. This theme was informed by three sub themes, namely; mentorship and sequencing of the programme, inadequate resources and equipment and demanding and Tedious.

#### Mentorship and Sequencing of Programme

This subtheme “Mentorship and sequencing of programme” reflected the negative experiences with regards to receiving mentorship from the supervisors either in the clinical placement areas or from the university faculty. It also reflected the feelings the students had with regards to sequencing of the courses in the programme.

With regards to sequencing of the courses learnt in class before exposure to the practice sites, most of the participants reported that most content covered in class was hard to apply in the clinical area as it was too general. They had learnt leadership and management and Health assessment and diagnostic reasoning as preparatory courses. However, the content covered in Health assessment and diagnostic reasoning was for adults and not paediatrics which was challenging to now practice on the paediatrics.

“I had knowledge in health assessment for adults for but no knowledge on how to assess a paediatric which was somehow embarrassing on the NICU when the doctors are asking questions”.

Still on sequencing of the programme, most participants reported that they were straight away exposed to the complicated condition without starting with the non-complicated ones. They stated that they initially thought they will start with managing the non-complicated cases as they were gaining confidence then later proceed to complicated ones.

“In the first week of my allocation to the NICU I was expected to work on a baby who had hypoxic Ischaemic encephalopathy. I felt inferior to the people who were there because I was not able to do it as I had not yet gained the expertise”.

The issue of working in this manner posed a challenge for the participants and most complained that before they could even get properly oriented to the environment, they had already started managing patients with complications.

Some of the participants reported that it was hard to work in the neonatal intensive care unit (NICU) because they were not midwives who are exposed to the NICU and had no prior knowledge or exposure but were ultimately expected to perform at the same level as those who had prior exposure to the NICU because they were all postgraduate students. They lamented that at undergraduate level they had a general bachelor’s degrees in nursing with minimal exposure to the NICU while others had never worked in NICU upon completion of their programme.

“I really felt under pressure to catch up with my colleagues who had worked in NICU for a long time. It felt like I had to work 10 times more for me to be able to be at the same level with them”.

Another area of concern which the participants noted was the lack of preceptors in the practice area in the early phase as they started the practice. They expressed disappoint at the issue of not having any preceptor in the clinical area because there was no one to mentor or guide them as they practiced. They felt mentors needed to provide guidance, support and feedback to enhance their learning experience. They also reported that even though the clinical area had no preceptor’s, faculty members from the University should have been checking on them.

Additionally, participants stated that most of what an individual aspires to be is what they see, hence it was hard to envision who they will become once they graduated. They stated that in the initial phase, nursing care of the neonate was taught by the midwives and doctors which resulted in some knowledge gaps during their clinical practice. However, towards the third term a neonatology nurse practitioner from the USA was engaged who not only helped them with specific aspects of neonatal nursing care but also changed their perception on their role as post graduate neonatal nurses and further explained the scope of practice.

“Doctors explained the pathophysiology so well but the nursing aspect of care was lacking”.

#### Demanding and Tedious

The subtheme “demanding and tedious” reflected the students’ experiences with regards to time allocation for the programme. Most of the participants reported that the programme was tiring because they were having clinical sessions during the day and theory classes in the evening. They reported that during the day it was busy as they were attending to patients and working with the doctors on the case study presentations. They felt the process of learning in the evening and attending to patients during the day made them tired and sometimes would not concentrate in the evening classes.

“I remember having a busy day in the NICU with a lot of babies with complicated cases. I finished quiet late and only rested for 15 minutes before the next class and I dozed the all-time I was in class”.

Most of the participants felt that it would be better to have the class and practice alternate on different days. They reported that it will give them time to think through and reflect on what they had learnt or seen in practice. On the other hand, the participants appreciated the idea of learning and practising at the same time. Some of them reported that they felt they could not afford to miss because it meant they will be behind.

“I was afraid to miss either class or clinical practice because I needed to learn as much as I can. I also noticed that during this period I studied more for me to understand the conditions I was likely to find in the ward”.

Additionally, the participants felt that the programme was tedious because most of the staff members took it for granted that because the students were in-service they already knew what was expected and would easily find their way around in the clinical setting. However, even if they already had ideas, most participants felt they needed some preparation as they were expected to practice on a higher level.

“I feel priority was given to classroom learning because most lecturers thought it was easier for us to find our way in any clinical setting”.

#### Inadequate Resources and Equipment

The subtheme “inadequate resources and equipment” reflected the challenges faced with provision of resources and the availability of equipment. Most of the participants reported the there was a lack of resources to be used in performing daily procedures during patient care.

“I had a baby who was in dire need of surfactant after I did my assessment but the hospital had no surfactant, so in this case I had to improvise”.

In the quest to acquiring knowledge, post graduate students are expected to be good both in the theory and practice. Most of the participants reported that they were unable to see and use a number of equipment they had learnt about in the clinical practice sites.

“I noticed we learnt about use of c-pap and saw pictures online, but when I went to the NICU the c-pap I found was not the ideal one it was an improvised version”.

Additionally, the participants reported concerns with the inability to see the actual equipment needed to be used in the clinical setting. They felt disadvantaged when they compare themselves with others in settings where equipment is available.

### 3.3. Theme 3: Favourable Educational Learning

The theme “favourable educational learning” reflected the positive experiences the students hard with the EECE model. It was informed by two subthemes namely; easy integration of theory to practice and supportive medical staff.

#### Easy Integration of Theory to Practice

The subtheme “easy integration of theory to practice” reflected the positive experiences of how early enhancement of clinical practice made theory to practice linkage easy.

Most of the participants reported that after exposure to the model a number of issues were clarified and things became clear. They reported that after using the model it became easy for them to integrate the knowledge gained in theory to Practice. Additionally, most of the participants reported that after exposure they were able to relate the information learnt in the basic sciences and link it to practice. According to the participants the clear explanations of the pathophysiology of different conditions made it easy for them to fully understand why they needed to take certain interventions which were exciting.

“I felt happy knowing that I know the condition fully and am able to understand the reasons why I am taking the interventions”.

Further, the participants stated that they enjoyed their experience because it helped improve their clinical skills. They stated that they did not need to observe others do procedures or assess patients but were fully involved in the care of patients. They stated that it is a hands-on experience which is what they needed. They expressed excitement at the thought of knowing that they are able to respond to questions and queries from an informed point of view both in theory and practice as they were now well vested in both areas.

#### Supportive Medical Staff

The subtheme “Supportive medical staff” reflected the role medical practitioners played in ensuring the practice area for the students was favourable. Most of the participants reported that unlike the hostile treatment they received from the nurses, the doctors were very receptive to them. They reported that the doctors went out of their way in providing guidance and spent a lot of time conducting beside teaching and listening to student’s case presentations. The doctors provided guidance and gave feedback immediately after the students presented.

“Feedback came was given there and then and this made it easy for me to know where I made mistakes and areas I needed to improve on”.

They stated that the support received made it easy to improve in the clinical skills as the programme was hands on.

### 3.4. Theme 4: Enhancing Personal and Professional Growth

The second theme “Enhancing personal and professional growth” explicated the changes brought about by use of EECE model. Most of the participants reported that they appreciated the learning experience as it helped them see things in a different perspective. The theme was informed by two subthemes, namely, promoting deeper learning and giving practice a purpose.

#### Promoting Deeper Learning

The subtheme “promoting deeper learning” reflected the practices which contributed to students understanding the content better. It is thought that deeper learning will help the students to survive and succeed in this twenty first century. Therefore, practices were used which promoted deep learning in the students.

Most of the participants reported that for them to have a deeper understanding of the conditions found in the NICU and the best methods of management the students were encouraged to personalise their learning and put in their best in order to achieve their goals. They were encouraged to use bedside learning where they interacted with the patients at length and got to have a deeper understanding of the conditions, secondly, they used the case study presentations. These presentations were done using the SBAR technique where they looked at the Situation, Background, Assessment and Recommendation. With this, the students were able to thoroughly assess the patients and give appropriate recommendations. The students were also able to receive feedback there and then as they presented the cases. This is how two of them expressed themselves:

“It was my first time using the SBAR technique but I enjoyed using it and it really help improve my way of assessing patients’ conditions”.

“What I enjoyed most about this learning is that I was able to get feedback there and then after case presentations”.

Additionally, the participants noted with pleasure that the learning approaches used contributed to them using critical thinking skills. They stated that they were able to assess the patients and make decisions on what should be done for the patients, the nursing interventions, the treatment to be received and the follow up care that should be implemented. One participant expressed herself saying:

“The practice made me to be able to think fast and make decisions”.

The participants also reported that presenting on a regular basis improved their communication skills and boosted their confidence at individual level. They also reported that it helped them to read a lot and do a lot of research before presenting their cases.

#### Giving Practice a Purpose

The theme “Giving practice a purpose” reflected the contentment students felt upon completion of the programme. Most participants stated that at the beginning of the programme they were not able to understand most things. However, towards the end things become much clearer and there was synchrony between the theory and the Practical. Further, they stated that as they familiarised themselves with the model it became easier to integrate what they had learnt in the basic sciences with the work they needed to do on the actual patients. They also stated that it was exciting to be able to learn and see the patients with the conditions you are from learning about there and then. This is how one of them expressed themselves:

“In the development of the lungs in neonates, I was able to appreciate and know at what stage each individual neonate was at”.

In addition, the learning experience also contributed to promotion of evidence-based practice. Participants reported that it was easy for them to notice methods that were working and those not working and come up with new solutions to problems. They also stated that there was no need to continue with practices that were not working which contributed to students participating in finding new ways of managing the conditions. This would not have been the case if the students did not spend more time in practice.

Lastly, the participants reported that the knowledge gained helped them to assess, diagnose, and be able to treat different conditions found in the wards. This eventually, lead to them gaining experience, knowledge, and exposure and helped us to grow professionally.

## 4. Discussion

Early and Enhanced Clinical Exposure (EECE) has been proposed as a means to improve the quality of nursing education at the University of Zambia and prepare postgraduate students for real-world clinical practice. These learning experiences reflected perceptions of individual postgraduate nursing students pursuing neonatal nursing as a result of having undergone the EECE model in their training. The theme, “learning experience” aimed to explore the experiences of postgraduate students with Early and Enhanced Clinical Exposure, with the goal of providing insights into both the positive and negative experiences students had with regards to the use of this model. This theme will be discussed together with two (2) other subthemes, favourable educational experience as well as challenging and hectic experience. Favourable Educational Experience

One of the key findings of studies on Early and Enhanced Clinical Exposure (EECE) is that it positively impacts the learning experience of postgraduate students. Through EECE, students were given opportunities to develop clinical skills and competencies in a real-world setting. This allowed them to apply the knowledge and theories learned in the classroom to actual patients, which was a valuable learning experience. This finding corroborates with a study conducted by [11], in which postgraduate students who received EECE reported a more positive learning experience than those who did not. The authors noted that EECE provided students with a safe environment to learn and make mistakes, and allowed for immediate feedback and support from clinical supervisors. The findings are also consistent with other studies on medical students by [6] and [12] [13] that have found that early clinical exposure can enhance the learning experience of students.

Another key finding of the current study was that it helped students integrate theoretical knowledge into practice. This outcome, as highlighted by [3], is one of the core principles of the EECE model which aims to provide clinical context to learning. This study outcome is in tandem with the findings of by [7] which revealed that the model helped the students to not only understand topics better than lecture classes, but also to apply this knowledge in clinical settings. It is also consistent with the findings of [12] [13] study which revealed that EECE enabled students to obtain a better and deeper understanding of medicinal theory and practice through the application of their knowledge in real hospital situations.

Most students reported receiving invaluable support of the medical doctors in terms of learning pathophysiology and treatment of neonatal conditions on the actual cases. They were particularly motivated that doctors not only spent time listening to their case studies, but also provided mentorship and guidance. This study finding is congruent with recommendations from studies by [1] which explained that the success of the EECE model was largely dependent on the support of other clinicians’ especially medical doctors.

The major concern for most students using the EECE model was sequencing of programs. The curriculum itself does not give students freedom to learn content in whichever order but to follow a laid down structure of courses and topics. This emerged as a serious challenge for most students as content covered in class was either too general or hard to apply in the clinical area. This outcome validates the findings of earlier studies by [14] which stated that for EECE to be effective, the cases should be discussed with students, and their context be linked to what was previously taught in the classroom. To ensure that students comprehend the significance of the basic sciences, the [3] recommends that it would be ideal to structure all teaching and learning sessions around a clinical scenario.

Lack of preceptors was another factor that contributed to the hectic and challenging outcome of students. This was a problem for most students in that they expected preceptors or even faculty members to be on the ward to provide guidance, support and feedback. Clinical education, provided by qualified preceptors, gives students the practical experience they need to succeed as advanced practice nurses. The preceptor’s primary task, arguably, is to socialize the student to the role of the nurse practitioner as a health care provider through both official and informal instruction [15]. In addition, [16] states that preceptors are an important component of clinical education because they provide students with exposure to their future roles as nurses.

Improving personal and professional progress” described the modifications made by using the EECE model. Students reported experiencing deeper learning and made sense of clinical practice. Earlier research demonstrated that EECE helped students comprehend their duties and increased their excitement and motivation for their clinical education [17]. Students reported having a deeper understanding of NICU conditions, case presentation, and utilization of the SBAR technique easier and contributed the utilization of critical thinking skills. The learning experience also contributed to promotion of evidence based practice as knowledge gained helped them to assess, diagnose, and treat different conditions, notice methods that were working and those not working and come up with new solutions to problems. This eventually, lead to them gaining interest experience, knowledge, exposure and interest and helped them to grow professionally. These finding are in accordance with a study conducted by [7] in the educational interventions in ECE was preferable regardless of their level of clinical exposure. About 91.5% of respondents who had any clinical experience concurred that ECE is superior to lecture sessions for grasping issues. The students were positive about EECE and were full of enthusiasm [18] and [19]. It is evident from the findings that EECE has a significant impact on learning, as evidenced by the students’ increased skill levels and assessments of potential use in their usual clinical practice.

While the study has provided the latest data on the actual experiences of postgraduate students with EECE model in Zambia, it is not without any limitations. The first limitation is one common with phenomenological studies were establishing the reliability and validity of the methods can be challenging [20]. Furthermore, because this study used a small cohort, it is difficult to generalize the findings.

## 5. Conclusion

The model has a significant impact on motivation to learn, as reported by the students’ increased skill level with potential for use in clinical practice. The model allowed for a safe environment to learn and make mistakes and allowed for immediate feedback and support from clinical supervisors. It also contributed to the promotion of evidence-based practice and critical thinking skills as knowledge gained helped to assess, diagnose, and treat different conditions. For the model to be more effective in achieving the desired outcomes of hands-on clinical practice, there is a need for more coordination to better the teaching faculty and clinical staff and placement of preceptors to enable students to have more support mentorship and allow for the application of theory into practice. Despite the limitations and challenges faced by the first cohort of students under the EECE model at UNZASoN, it was recommended that the model be implemented in all the postgraduate programs for full-time students as its benefits outweighed the challenges.

## Figures and Tables

**Table 1.** Themes.

THEME	CATEGORY	SUB-CATEGORY

1) Identity and role confusion	Identity confusion	• Poor description of tasks • Mismatch between expectations and tasks • Hostile nursing staff behaviour
	Emotional outburst	• Fear • Stress • Anxiety

2) Challenging and hectic experience	• Mentorship and supervision	• No preceptors on the wards • No guidance • Faculty did not usually pass through to check
	• Sequencing of programme	• Mismatch between courses learnt in class and those in clinical area • Starting it complicated cases then non complicated
	• Demanding and Tedious	• Tiring • No time to rest and reflect • You cannot afford to miss a day without studying
	• Inadequate resources and equipment’s and materials	• Not enough materials to use on daily routines • Not enough resources needed to care for nursing care

3) Favourable Educational experience	• Easy integration of knowledge from theory and practice	• Able to link theory to practice • Able to link basic sciences to practice
	• Supportive medical staff	•

4) Enhancing personal and professional growth	Promoting deeper learning	• Bedside learning and presentation of case studies • Encouraged critical thinking • Practicing patient care using SBAR technique
	Giving practice a purpose	• Integration of Basic Sciences • Promoting evidence-based practice
